# Androgen signalling in the ovaries and endometrium

**DOI:** 10.1093/molehr/gaad017

**Published:** 2023-05-12

**Authors:** Abbey C Lissaman, Jane E Girling, Lynsey M Cree, Rebecca E Campbell, Anna P Ponnampalam

**Affiliations:** Department of Physiology, Faculty of Medical and Health Sciences, University of Auckland, Auckland, New Zealand; Department of Anatomy, School of Biomedical Sciences, University of Otago, Dunedin, New Zealand; Department of Obstetrics and Gynaecology, University of Auckland, Auckland, New Zealand; Department of Physiology and Centre for Neuroendocrinology, University of Otago, Dunedin, New Zealand; Department of Physiology, Faculty of Medical and Health Sciences, University of Auckland, Auckland, New Zealand; Pūtahi Manawa-Healthy Hearts for Aotearoa New Zealand, Centre of Research Excellence, New Zealand

**Keywords:** androgens, steroid hormones, receptor, testosterone, estrogen, progesterone, female, endometrium, ovaries

## Abstract

Currently, our understanding of hormonal regulation within the female reproductive system is largely based on our knowledge of estrogen and progesterone signalling. However, while the important functions of androgens in male physiology are well known, it is also recognized that androgens play critical roles in the female reproductive system. Further, androgen signalling is altered in a variety of gynaecological conditions, including endometriosis and polycystic ovary syndrome, indicative of regulatory roles in endometrial and ovarian function. Co-regulatory mechanisms exist between different androgens, estrogens, and progesterone, resulting in a complex network of steroid hormone interactions. Evidence from animal knockout studies, *in vitro* experiments, and human data indicate that androgen receptor expression is cell-specific and menstrual cycle stage-dependent, with important regulatory roles in the menstrual cycle, endometrial biology, and follicular development in the ovaries. This review will discuss the expression and co-regulatory interactions of androgen receptors, highlighting the complexity of the androgen signalling pathway in the endometrium and ovaries, and the synthesis of androgens from additional alternative pathways previously disregarded as male-specific. Moreover, it will illustrate the challenges faced when studying androgens in female biology, and the need for a more in-depth, integrative view of androgen metabolism and signalling in the female reproductive system.

## Introduction

While androgens have historically been prioritized in the context of male physiology, we now have a better understanding of the important roles that they play in the female reproductive system (Gibson *et al.*, 2014; Prizant *et al.*, 2014; Walters, 2015; Walters *et al.*, 2016; Simitsidellis *et al.*, 2018). However, there is still a significant gap in our knowledge when it comes to the roles of specific androgens, receptor interactions, and regulation of androgen signalling, particularly in female organ systems and in female physiology. Androgens, which include dehydroepiandrosterone (DHEA), androstenedione, testosterone and dihydrotestosterone (DHT), signal via androgen receptors (ARs), which are expressed in many tissues including the brain, muscle, liver, breast, ovaries, and endometrium (Gibson *et al.*, 2014; Uhlén *et al.*, 2015). Evidence suggests that, like estrogen and progesterone receptors, AR expression in the female reproductive tract is cyclic and cell and tissue-specific (Mertens *et al.*, 2001; Walters *et al.*, 2016; Gibson *et al.*, 2018a), indicating that androgens are an important and tightly regulated facet of female reproduction (Simitsidellis *et al.*, 2018; Gibson *et al.*, 2018a). The heterogeneous, tissue-specific expression of ARs, their multifaceted interactions with each other and associated regulatory factors, and limitations in our ability to quantify androgen levels and activity, add additional challenges when evaluating androgen actions in female reproductive biology. Combined with prior assumptions that androgens are more important in male biology, these factors have perhaps contributed to the lack of progress in this research field.

Bringing together findings from animal studies, *in vitro* experiments, and data from patients with polycystic ovary syndrome (PCOS) and other conditions involving altered androgen signalling, this review will outline the current understanding of androgen synthesis in females (defined as individuals with a phenotype consistent with female, born with a uterus and ovaries). This review will discuss key androgen types, classical and non-classical androgen synthesis pathways, interactions between androgens and estrogens, progesterone, and their receptors, and how hormone co-regulation influences key processes in the ovary and endometrium. Androgen signalling in other female reproductive tissues, such as the fallopian tubes, breast, and brain, has been described elsewhere and will not be the focus of this review (Kensler *et al.*, 2018; Kight and McCarthy, 2020; Maclean *et al.*, 2020; Rehbein *et al.*, 2021).

## Androgen synthesis pathways

**Figure 1.**
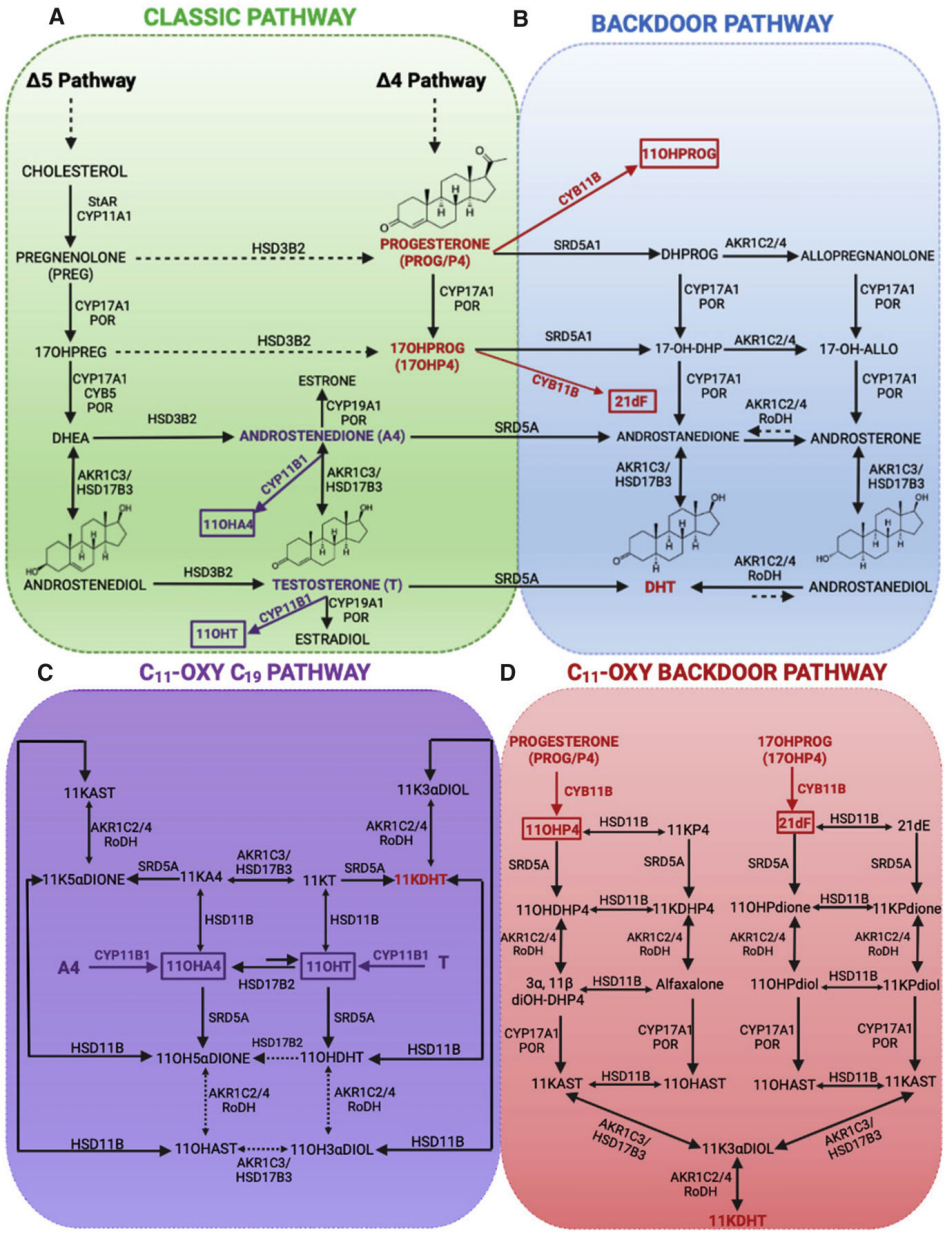
**Androgen synthesis pathways in the female reproductive system.** Conversion of cholesterol-derived progesterone to androgens, and androgens to estrogens, by steroidogenic hormones expressed by adrenal and ovarian tissues, via the (**A**) classical, (**B**) backdoor, (**C**) c11-oxy, and (**D**) c11-oxy backdoor pathways. 11KA4, 11-ketoandrostenedione; 11KDHT, 11-ketodihydrotestosterone; 11KT, 11-ketotestosterone; 11OHA4, 11 beta-hydroxyandrostenedione; 11OHP4, 11 beta-hydroxyprogesterone; 11OHT, 11 beta-hydroxytestosterone; 21dF, 21-deoxycortisol; AKR, aldoketo reductases; CYP, cytochrome p450 enzymes; DHEA, dehydroepiandrosterone; DHT, dihydrotestosterone; HSD, hydroxysteroid dehydrogenases; POR, cytochrome P450 oxidoreductase; SRD5A, 5-alpha reductase; StAR, steroidogenic acute regulatory protein. Reprinted with permissions from Naamneh Elzenaty *et al.* (2022).

There are presently four known androgen synthesis pathways, which have been summarised in Fig. 1. In females, androgens are produced primarily by the so-called classical (Fig. 1A) and backdoor (Fig. 1B) pathways, typical of the ovary, and also by the c11-oxy (Fig. 1C), and c11-oxy backdoor (Fig. 1D) pathways, typical of the adrenal glands (Naamneh Elzenaty *et al.*, 2022). Androgen synthesis also occurs in peripheral or target tissues, such as the endometrium, which expresses a number of steroidogenic enzymes, including 3 beta-hydroxysteroid dehydrogenase (3β-HSD) and 17 beta-hydroxysteroid dehydrogenases (17β-HSD1 and 2) (Cloke and Christian, 2012; Labrie *et al.*, 2017). The classical pathway of androgen production (Fig. 1A) involves metabolism of cholesterol to progesterone, androgen precursors—DHEA and androstenedione—and key androgen subtypes—testosterone (which can be further converted to estrogens), and the more potent form DHT (Hillier *et al.*, 1997; Burger, 2002). Other androgen types, such as 11-ketoandrostenedione (11KA4), 11-ketotestosterone (11KT), and 11-ketodihydrotestoterone (11KDHT), have been identified in the circulation but are not yet widely measured or used clinically owing to the lack of efficient techniques for detection (Schiffer *et al.*, 2018; Yu *et al.*, 2022).

The initial rate-limiting cholesterol transfer step is mediated by steroidogenic acute regulatory protein (StAR) (Jakimiuk *et al.*, 2001; Sander *et al.*, 2011), the expression of which promotes testosterone and DHT production via DHEA and androstenedione in the follicular phase, and progesterone production in the luteal phase (Devoto *et al.*, 2002; Men *et al.*, 2017). Cholesterol mobilization and metabolism is also regulated by translocator proteins (Costa *et al.*, 2018), and cholesterol side chain cleavage enzyme, CYP11A, expressed by theca cells (Wickenheisser *et al.*, 2012). For a more comprehensive review on these steroidogenic enzymes, and associated co-regulator proteins, we recommend referring to some more detailed recent reviews (Kempná *et al.*, 2015; Miller, 2017; Naamneh Elzenaty *et al.*, 2022).

More recent investigations have identified an alternative ‘backdoor’ pathway of androgen synthesis in addition to the ‘classical’ pathway (Fig. 1B) (Auchus, 2004; Miller and Auchus, 2011; Marti *et al.*, 2017). This pathway relies on the reduction of 17OH-progesterone by 5α-reductase and 3β-HSD1 and 3β-HSD3 (encoded by aldo-keto reductase family 1 members C2 and C4 (AKR1C2/4)), the subsequent intermediate reactions catalysed by steroid 17 alpha-hydroxylase/17,20 lyase (cytochrome P450c17/CYP17) and 17β-HSD5, and an oxidation step to the potent androgen DHT by 17β-HSD6 (Lee and Kim, 2022) (Fig. 1B). Crucially, the backdoor pathway bypasses the intermediate steroids involved in the classical pathway—DHEA, androstenedione, and testosterone (Lee and Kim, 2022).

While occurring primarily in the male testes and predominantly known for roles in normal prenatal masculinization (Miller and Auchus, 2019; O’Shaughnessy *et al.*, 2019), the backdoor pathway may also occur in females, particularly in cases of abnormal androgen synthesis. Although not well explored in female biology, this backdoor pathway has been linked to PCOS (O’Reilly *et al.*, 2014; Marti *et al.*, 2017; Kempegowda *et al.*, 2020; Lee and Kim, 2022), P450c21 deficiency, and congenital adrenal hyperplasia (Reisch *et al.*, 2019; Lee and Kim, 2022). How this pathway is regulated in normal female reproductive physiology has not been studied in much detail. However, expression of aldo-keto reductases and 5α reductases has been observed in the normal human ovary (Marti *et al.*, 2017).

Another alternative pathway of androgen synthesis and metabolism has also been recently described (Swart and Storbeck, 2015; Pretorius *et al.*, 2017; Naamneh Elzenaty *et al.*, 2022). The c11-oxy pathway (Fig. 1C) is a non-classical androgen synthesis pathway, and an important source of adrenal-derived androgens, specifically the potent and bio-active 11-oxy androgens. In this pathway, androstenedione and testosterone from the classical pathway are converted by CYP11B1 (11β-hydroxylation). Androstenedione is converted to 11β-hydroxyandrostenedione (11OHA4), and testosterone is converted to 11-hydroxytestosterone (11OHT), which are then further metabolised to 11KA4 and 11KT (Naamneh Elzenaty *et al.*, 2022) (Fig. 1C).

A fourth pathway, the ‘c11-oxy backdoor’ or non-classical backdoor pathway (Fig. 1D), uses 11OHP4 and 21dF, produced in the classical backdoor pathway via 11b-hydroxylation (CYP11B) of progesterone and 17OH-progesterone in the synthesis of 11KDHT, using classical pathway enzymes (Pretorius *et al.*, 2017; Naamneh Elzenaty *et al.*, 2022). 11KT and 11KDHT produced from these pathways have high potencies equivalent to testosterone, and are powerful AR agonists (Pretorius *et al.*, 2017; Naamneh Elzenaty *et al.*, 2022). While these c11-oxy androgen subtypes are produced in the adrenal glands, expression of some enzymes, such as 11β-HSD2, 17β-HSD5 (also known as AKR1C3), and 5α-reductase, that facilitate the production of 11KDHT, have also been identified in human endometrial tissues (McDonald *et al.*, 2006; Tanaka *et al.*, 2015; Konings *et al.*, 2018; Hojnik *et al.*, 2020). Although this pathway is not yet well-studied outside of the adrenal gland, particularly in female tissues, the expression of these enzymes may be indicative of local c11-oxy androgen metabolism in the endometrium.

## Androgen subtypes and their functions

There is substantial published data on the four main androgen subtypes—DHEA, androstenedione, testosterone, and DHT (Lee and Kim, 2022; Naamneh Elzenaty *et al.*, 2022). Their key effects in the female reproductive tract have been summarized in Table 1.

**Table 1.** A summary of key androgen subtypes in the context of female reproductive physiology.

Androgen	Produced	Circulating levels	Receptor interactions	Relative potency towards AR	Effects	Altered in disease	References
Dehydroepiandrosterone (DHEA)	Adrenal zona reticularis, ovarian theca cells, also converted to/from DHEAS	Largely constant	AR (weak), ERβ (strong), PPAR	+	Oocyte maturation, oocyte metabolism, follicle viability, endometrial receptivity, decidualization	Used to induce PCOS effects in mice, elevated in PCOS	Benjamin *et al.* (2021), Bracho *et al.* (2020), Çelik *et al.* (2017), Chern *et al.* (2018), Chimote and Chimote (2018), Corrie *et al.* (2021), Dhayat *et al.* (2018), Gibson *et al.* (2018a,b), Kamada *et al.* (2022), Labrie *et al.* (2017), Lv *et al.* (2022), Mahmoud *et al.* (2018), Piltonen *et al.* (2002), Prough *et al.* (2016), Qin *et al.* (2016), Salonia *et al.* (2008), Schwarze *et al.* (2018), Stener-Victorin *et al.* (2020), and Vojnović Milutinović *et al.* (2021)
Androstenedione	Adrenal zona fasiculata, ovarian stroma, or intracellularly from DHEA	Mainly bound by SHBG. Highest right before ovulation, circadian variation.	AR	++	Ovarian response, follicular growth/development, follicle viability	Elevated in PCOS	Bleach *et al.* (2018), Burduli *et al.* (2020), Burger (2002), Georgopoulos *et al.* (2014), Hausknecht *et al.* (1982), Hilborn *et al.* (2017), Horie *et al.* (1992), Labrie *et al.* (2017), Lima-Verde *et al.* (2010), Lv *et al.* (2022), Mahmoud *et al.* (2018), Piltonen *et al.* (2002), Salonia *et al.* (2008), and Vermeulen and Verdonck (1976)
Testosterone	Adrenal zona fasiculata, ovarian stroma, and from androstenedione	Mainly bound by SHBG. Mid-cycle peak, highest right before ovulation, lowest early proliferative/late secretory	AR	++++	Promote IGF-1 expression, oocyte metabolism, follicular growth/maturation, excess: endometrial atrophy, reduced cell proliferation	Elevated in PCOS (incl prenatally), reduced in endometriosis (incl prenatally)	Abbott *et al.* (2019), Atukorala *et al.* (2022), Braunstein *et al.* (2011), Dawood and Saxena (1976), Dinsdale *et al.* (2021), Franik *et al.* (2019), Grassi *et al.* (2020), Krüger *et al.* (2023), Laird *et al.* (2017), Løssl *et al.* (2020), Loverro *et al.* (2016), Lv *et al.* (2022), Noventa *et al.* (2019), Perrone *et al.* (2009), Rothman *et al.* (2011), Vermeulen and Verdonck (1976), Wyskida *et al.* (2017), and Zhou *et al.* (2017)
Dihydrotestosterone (DHT)	Peripherally from testosterone, or via the backdoor pathway from androsterone. Some from adrenal zona fasiculata	Mainly bound by SHBG. Low circulating levels, small rise around ovulation	AR (2x affinity of testosterone, ERα, ERβ	++++++	Decidualization, regulation of cell stress and apoptosis, endometrial gland development	Used to induce PCOS effects in mice, elevated in PCOS, elevated in endometrial cancers	Auchus (2004), Cárdenas and Pope (2002, 2004), Cheon *et al.* (2009), Chin *et al.* (2015), Dawood and Saxena (1976), Duda *et al.* (2017), Grino *et al.* (1990), Hung and Gibbons (1983), Kajihara *et al.* (2014), Laird *et al.* (2017), Laurent *et al.* (2016), Lee and Kim (2022), Lv *et al.* (2022), Marshall *et al.* (2011), Marti *et al.* (2017), Murray *et al.* (1998), Nantermet *et al.* (2005), Rothman *et al.* (2011), Stener-Victorin *et al.* (2020), Tanaka *et al.* (2015), Vermeulen and Verdonck (1976), and Vojnović Milutinović *et al.* (2021)

AR, androgen receptor; DHEA, dehydroepiandrosterone; DHEAS, dehydroepiandrosterone sulfate; DHT, dihydrotestosterone; ERα, estrogen receptor alpha; ERβ, estrogen receptor beta; IGF-1, insulin-like growth factor 1; PPAR, peroxisome proliferator-activated receptor; SHBG: steroid hormone-binding globulin; + indicates relative potency of each subtype towards AR, in comparison to testosterone.

It should be noted that recent studies using mass spectrometry have identified other androgens that circulate in the female body, including some produced in the aforementioned c11-oxy synthesis pathway and associated backdoor pathway, such as 11KT, and 11OHT (Grassi *et al.*, 2020; Yu *et al.*, 2022). Our understanding of androgens in female physiology, particularly androgen subtypes more recently identified, may be in part limited by the detection methods used to identify and measure these hormones; liquid chromatography–tandem mass spectrometry is more accurate and specific than traditional immunoassays used in a lot of earlier literature (Demers, 2010; Tosi *et al.*, 2016). Although these androgen subtypes appear to contribute only a small amount to the total measurable androgens in circulation, we expect that further research will reveal important physiological roles. While the remainder of this review will focus on the four more commonly known androgen types, the potential contributions of these other recently identified androgen subtypes should not be ignored.

## Androgen receptors

The main receptors that androgens bind to and exert their effects through are summarized in Table 2.

**Table 2.** Key steroid hormone receptors involved in androgen signalling in female reproductive physiology.

Receptor	Receptor family	Gene	Tissue expression	Ligands	Signalling	References
AR	Ligand-dependent nuclear transcription factors	*AR* gene, X chromosome	Brain Muscle Liver Breast Ovary Endometrium	Androstenedione, testosterone, DHT, and most 11-oxy androgens, e.g. 11KT ^ and 11KDHT ^	Classical/DNA binding-dependent mechanism via AREs, non-DNA binding mechanism via second messenger pathways	Chang *et al.* (1995), Davey and Grossmann (2016), Gibson *et al.* (2014), Marcelli (2017), Quigley (1998), Roesler *et al.* (1990), Uhlén *et al.* (2015), and Yu *et al.* (2020)

ERα	Ligand-dependent nuclear transcription factors	*ESR1* gene, chromosome 6	Endometrium Ovary Breast	Estrogens, DHEA, DHT	Classical/DNA binding-dependent and estradiol-dependent, direct genomic signalling via EREs, e.g. feedback to alter ER/PR expression Non-classical non-DNA binding, estradiol-dependent, indirect genomic signalling via second messengers and other transcription factors Estrogen independent signalling	Cárdenas and Pope (2004), Chimote and Chimote (2018), Chin *et al.* (2015), Fuentes and Silveyra (2019), Gibson *et al.* (2018a,b), Klinge (2001), Paterni *et al.* (2014), Petz *et al.* (2004), Prough *et al.* (2016), Tang *et al.* (2019), and Yu *et al.* (2022)
ERβ	Ligand-dependent nuclear transcription factors	*ESR2* gene, chromosome 14	Cardiovascular system Nervous system Ovary Endometrium	Estrogens, DHEA^, DHT		

GPER	7-transmembrane G-protein coupled receptor (GPCR)	*GPER1*, chromosome 7	Endometrium Ovary Breast Brain Adrenal gland Kidney Heart Endothelium	Estradiol^	Non-genomic intracellular estrogen signalling	Luo and Liu (2020), Plante *et al.* (2012), Thomas *et al.* (2005), Yu *et al.* (2022)

PRA	Ligand-dependent nuclear transcription factors	*PGR*, chromosome 11	Endometrium Myometrium Ovary Mammary gland Brain Cardiovascular system Central nervous system	Progesterone	Classical/nuclear/genomic signalling via PR dimer binding to PREs, extranuclear/non-genomic signalling (membrane progesterone receptors)	Conneely and Lydon (2000), DeMayo and Lydon (2020), Gao *et al.* (2005), Grimm *et al.* (2016), Hu and Funder (2006), Matias *et al.* (2000), Mauvais-Jarvis *et al.* (2022), Nadal *et al.* (2017), and Taraborrelli (2015)
PRB						

11KDHT, 11-ketodihydrotestoterone; 11KT, 11-ketotestosterone; AR, androgen receptor; DHEA, dehydroepiandrosterone; DHT, dihydrotestosterone; ERα, estrogen receptor alpha; ERβ, estrogen receptor beta; ERE, estrogen response element; ESR1, estrogen receptor 1; ESR2, estrogen receptor 2; GPCR, G-protein coupled receptor; GPER, G-protein coupled estrogen receptor; PGR, progesterone receptor; PRA, progesterone receptor isoform A; PRB, progesterone receptor isoform B; PRE, progesterone response element. ^ indicates a particularly strong binding affinity.

**Figure 2.**
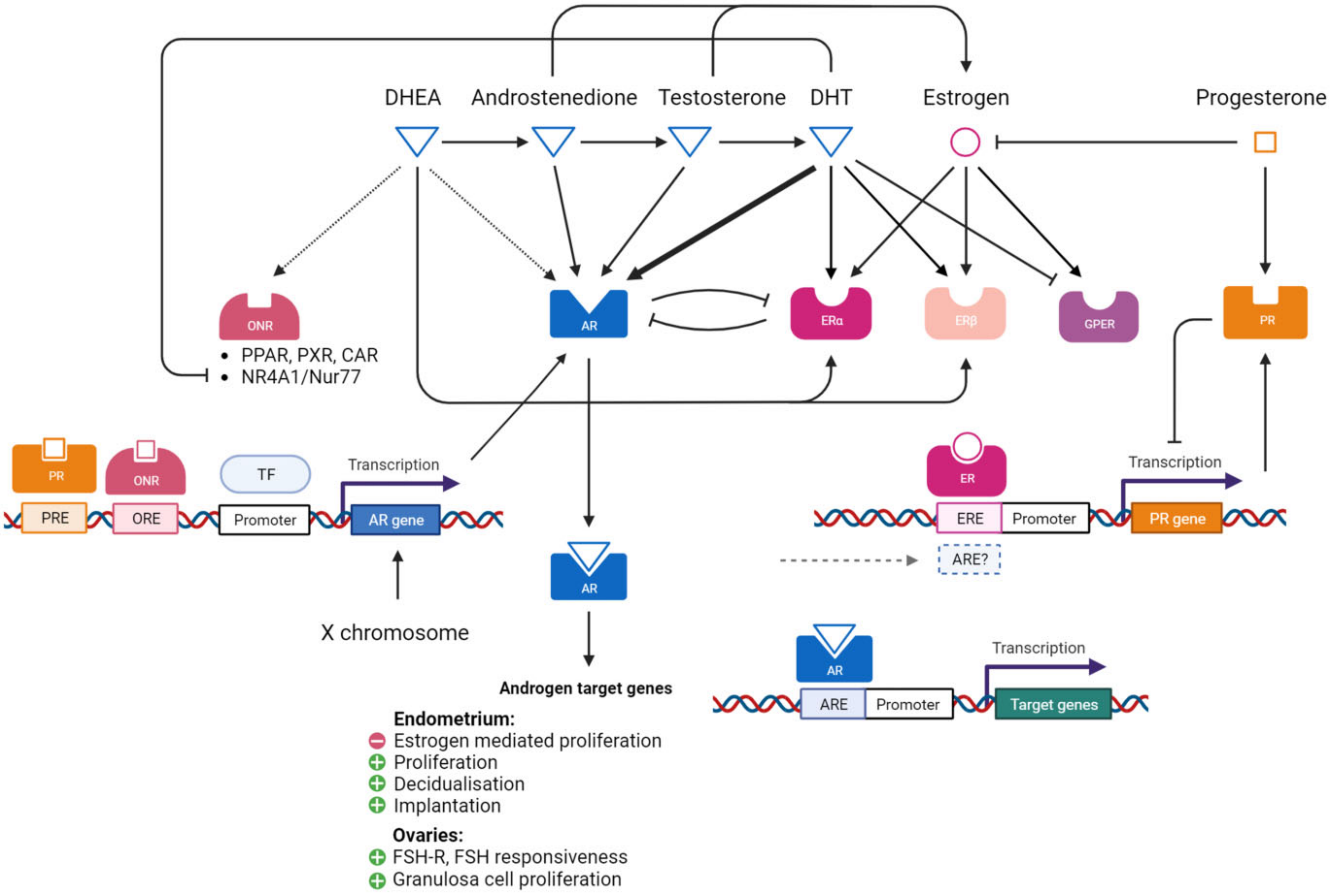
**Summary of key androgen signalling pathways and effectors, and interactions with other steroid hormone receptors in the female reproductive system.** The complex network of reproductive steroid hormones—estrogens, progesterone, and androgens—their interaction with receptors, and the co-regulatory interactions between them that co-ordinate their effects in the ovaries and endometrium is shown. Arrows between steroids denote conversion. Arrows between steroids and receptors denote binding; a thick arrow indicates high-affinity binding of DHT to AR compared to other androgens; similarly, a dotted arrow denotes a comparatively weak binding affinity of DHEA to AR. DHEA, dehydroepiandrosterone; DHT, dihydrotestosterone; AR, androgen receptor; ONR, orphan nuclear receptors; PPAR, peroxisome proliferator-activated receptor; PXR, pregnane X receptor; CAR, constitutive androstane receptor; NR4A1, nuclear receptor subfamily 4 group A member 1; ERα, estrogen receptor alpha; ERβ, estrogen receptor beta; GPER, G-protein-coupled estrogen receptor; PR, progesterone receptor; TF, transcription factor; ERE, estrogen response element; ARE, androgen response element; PRE, progesterone response element; ORE, orphan nuclear receptor response element. Figure created using BioRender.com.

Co-regulatory mechanisms and feedback actions of androgens, their metabolites, and receptors, are important in female reproduction. For instance, progesterone receptor (PR) antagonists have also been shown to enhance AR expression *in vitro* (Narvekar *et al.*, 2004), and androgen activity via ARs upregulate PR gene and protein expression in the endometrium (Babayev *et al.*, 2017) (Fig. 2). Furthermore, estrogen receptor (ER) α (*ER*α) expression can be downregulated by androgen signalling through AR to attenuate the effects of estrogen in the endometrium (Cárdenas and Pope, 2004). Similarly, DHT signalling via AR has been shown to downregulate the expression of recently-identified G-protein-coupled ERs (GPERs) in breast cancer cells, both at the gene and protein level (Shen *et al.*, 2017). GPERs are also expressed in the endometrium and ovaries of rodents (Otto *et al.*, 2009) and humans (Filardo and Thomas, 2012; Heublein *et al.*, 2012; Plante *et al.*, 2012), although their modulation by androgens and ARs is yet to be explored. Indeed, some research demonstrates altered endometrial GPER expression in patients with PCOS (Hulchiy *et al.*, 2016), which may indicate further crosstalk between ARs and GPERs.

Some androgens also bind to orphan nuclear receptors, including peroxisome proliferator-activated receptors (PPAR), pregnane X receptor, and constitutive androstane receptor (Prough *et al.*, 2016) (Fig. 2). DHEA, for instance, binds nuclear receptors with a relatively low affinity compared to either ARs or ERs (Prough *et al.*, 2016). In mice, signalling via the orphan nuclear receptor Nur77, which is expressed in theca cells and has been linked to follicular maturation, regulates the *AR* gene via a response element in the promoter region, and promotes AR expression in developing follicles (Dai *et al.*, 2012). Nur77 could be also downregulated by DHT, and its overexpression demonstrates a negative feedback mechanism that modulates androstenedione production and growth of preantral follicles (Xue *et al.*, 2012). Interestingly, the same receptor, known as nuclear receptor subfamily 4 group A member 1 (NR4A1) in humans, is expressed in human female reproductive tissues and has recently been linked to the regulation of endometrial receptivity and implantation (Cai *et al.*, 2023). With the exception of AR and ERs, the specific receptor interactions in androgen signalling have not been well explored in human female physiology.

## Androgens in ovarian physiology

### AR knockout models

ARs are expressed in both thecal and granulosa cells of the growing follicle (Horie *et al.*, 1992; Chang *et al.*, 2013; Franks and Hardy, 2018). The use of transgenic tools in animal models has elucidated the importance of androgen signalling in the ovary. Global androgen resistant AR knock out (ARKO) mouse models demonstrate a variety of abnormal ovarian effects including longer estrous cycles, fewer corpora lutea, impaired follicular development, increased follicular atresia, impaired oocyte retrieval, reduced fertilization capacity, and characteristics of premature ovarian insufficiency (Yeh *et al.*, 2002; Hu *et al.*, 2004; Shiina *et al.*, 2006; Walters *et al.*, 2007, 2009; Sen and Hammes, 2010; Zhou, 2010; Ma *et al.*, 2017). Follicular development can be inhibited via AR inhibition or knockout, and rescued by supplementation of non-aromatizable androgens (DHT) (Murray *et al.*, 1998; Sen and Hammes, 2010), even in the absence of estrogen (Wang *et al.*, 2001), demonstrating AR-dependent effects on ovarian function (Duda *et al.*, 2017). Global ARKOs also demonstrate deficiencies in neuroendocrine feedback mechanisms. Elevated FSH (Walters *et al.*, 2009) and impaired magnitude and timing of LH and estradiol peaks preceding ovulation (Cheng *et al.*, 2013) demonstrate a role for androgen feedback in modulating the hypothalamic–pituitary–gonadal (HPG) axis. Similarly, Cheng (2013) noted that follicles from ARKO mice may also be synthesizing less estrogen and, thus, effects may not exclusively be caused by impaired androgen activity (Cheng *et al.*, 2013). Cell and tissue-specific ARKO models have also been used to better understand specific AR actions. Sen and Hammes (2010) demonstrated that granulosa cell-specific ARKO mice developed a very similar ovarian phenotype to global knockouts, with significant reductions in ovarian function and fertility (Sen and Hammes 2010). Furthermore, many of the PCOS-like features associated with excess androgens, including impaired estrous cyclicity and insulin resistance, appear to be mediated by extra-ovarian ARs in neurons, adipocytes and the liver, as demonstrated by neuronal/granulosa and adipocyte specific knock out models (Cox *et al.*, 2020; Andrisse *et al.*, 2021).

### Follicular development

**Figure 3.**
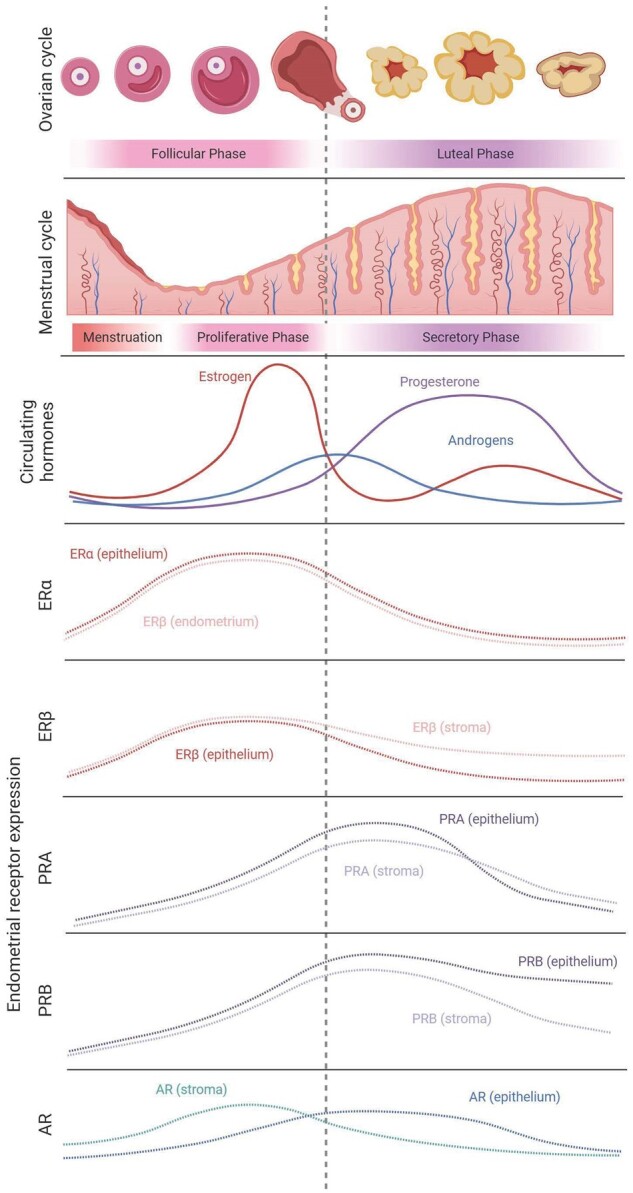
**Steroid hormones and receptor expression in the female reproductive system.** Summary of relative estrogen, progesterone, and total estimated androgen levels and their receptors in relation to the ovarian and menstrual cycles. ERα, estrogen receptor alpha; ERβ, estrogen receptor beta; PRA, progesterone receptor isoform A; PRB, progesterone receptor isoform B; AR, androgen receptor. Collated data used to estimate relative circulating hormone levels (Guerrero *et al.*, 1975; Dawood and Saxena, 1976; Vermeulen and Verdonck, 1976; Massafra *et al.*, 1999; Salonia *et al.*, 2008; Rothman *et al.*, 2011; Skiba *et al.*, 2019; Atukorala *et al.*, 2022; Krüger *et al.*, 2023) and relative receptor expression patterns in the endometrium (Mote *et al.*, 2000; Mertens *et al.*, 2001; Hapangama *et al.*, 2015; Gibson *et al.*, 2018a, 2014). No information could be found regarding specific receptor expressions in the ovary. Figure created using BioRender.com.

Androgen signalling has a key role throughout nearly all stages of follicular development (Walters *et al.*, 2016; Franks and Hardy, 2018). Some animal experiments demonstrate that AR expression in ovaries declines with follicular development (Hillier *et al.*, 1994; Tetsuka *et al.*, 1995; Cárdenas and Pope, 2004), while immunohistochemical analyses of human ovaries demonstrate increasing AR expression with follicular maturation (Horie *et al.*, 1992; Rice *et al.*, 2007), and high expression in granulosa cells immediately after ovulation (Horie *et al.*, 1992). This corresponds to a peak in circulating testosterone around the time of ovulation, and lowest levels in the early proliferative and late secretory stages of the menstrual cycle (Fig. 3) (Rothman *et al.*, 2011; Atukorala *et al.*, 2022; Krüger *et al.*, 2023).

In addition to binding AR, DHEA has a strong affinity for ERs, particularly ERβ, through which many of its effects are regulated (Chimote and Chimote, 2018), and a relatively low affinity for orphan nuclear receptors (Prough *et al.*, 2016). Through ERβ activation, DHEA influences oocyte maturation and calcium homeostasis, and, via the orphan nuclear receptor PPAR, influences mitochondrial activity, oxidative stress, and follicle quality and quantity (Prough *et al.*, 2016; Chimote and Chimote, 2018). In mice and in patients with poor ovarian response, DHEA supplementation following ovarian stimulation improved rates of oocyte retrieval (Chern *et al.*, 2018) and clinical pregnancy rate (Gleicher *et al.*, 2010; Schwarze *et al.*, 2018). Serum androstenedione levels may also be positively associated with oocyte retrieval and ovarian response (Burduli *et al.*, 2020), and *in vitro* studies have demonstrated a role for androstenedione and FSH in promoting follicular growth and development, and maintaining follicular viability and activation (Lima-Verde *et al.*, 2010). Similarly in rats, DHEA and androstenedione treatment influences ovarian follicle viability, activation, differentiation, and estrogen production (Mahmoud *et al.*, 2018) via FSH-mediated pathways (Hamel *et al.*, 2005; Lima-Verde *et al.*, 2010; Walters *et al.*, 2017).

Both testosterone and DHT promote insulin-like growth factor-1 expression and increase FSH receptor expression, with stimulatory effects on primordial follicle growth and maturation, oocyte metabolism, follicular recruitment, and oocyte retrieval (Rice *et al.*, 2007; Sen and Hammes, 2010; Laird *et al.*, 2017; Noventa *et al.*, 2019; Løssl *et al.*, 2020). Indeed, serum androgen levels tend to increase throughout follicular development, reaching a peak near ovulation (Atukorala *et al.*, 2022; Krüger *et al.*, 2023). Ligand-bound ARs increase the response of follicles to FSH and, furthermore, FSH exerts its own effect on steroidogenic enzymes, influencing androgen availability, and metabolism in granulosa cells via a feedback mechanism (Lenie and Smitz, 2009). In addition to potential improvements in ovarian function, high androgen concentrations may be antagonistic, which suggests there may be a threshold effect of androgens on follicular function (Zeleznik *et al.*, 2004).

Similarly, late in follicular development, androgens inhibit follicle growth and estrogen production, stimulating granulosa cell apoptosis, and a transition to follicular maturation (Walters *et al.*, 2008), as well as antral cavity development (Murray *et al.*, 1998). In addition, androgen signalling in the ovaries stimulates corpora lutea formation via the promotion of FSH receptor expression, thus also having an indirect effect on progesterone production (Gregoraszczuk, 1991; Carrizo *et al.*, 1994).

## Androgens in the endometrium

### AR expression in the endometrium and across the menstrual cycle

Some research suggests that AR expression in endometrial stromal and glandular cells is consistent across the menstrual cycle (Horie *et al.*, 1992). Other evidence indicates that in humans, ARs are predominantly expressed in stromal cells and elevated in the mid-to-late proliferative phase (Mertens *et al.*, 2001, 1996; Apparao *et al.*, 2002; Simitsidellis *et al.*, 2018). AR expression is comparatively lower in epithelial cells, with elevated AR expression in the late proliferative phase endometrial epithelium, maintained in the secretory phase (Fig. 3) (Apparao *et al.*, 2002; Gibson *et al.*, 2018a).

### Proliferation

ARKO mice (Choi *et al.*, 2015) and ovariectomized rodents (Nantermet *et al.*, 2005; Simitsidellis *et al.*, 2016) have been used to demonstrate associations between testosterone and DHT administration and endometrial cell proliferation, potentially through the regulation of epidermal growth factor signalling (Watson *et al.*, 1998). Other *in vitro* studies have instead demonstrated inhibitory effects of androstenedione, testosterone, and DHT on the proliferation of both human endometrial cell lines (Neulen *et al.*, 1987; Rose *et al.*, 1988; Marshall *et al.*, 2011; Park and Han, 2013), and isolated endometrial epithelial cells (Tuckerman *et al.*, 2000), antagonizing the proliferative effects of estrogen. In humans, treatment with exogenous testosterone above normal physiological levels for females demonstrates negative feedback effects, with endometrial atrophy and reduced cell proliferation (Miller *et al.*, 1986; Perrone *et al.*, 2009). The regulation of cell stress and apoptosis may also be androgen-dependent, with DHT treatment significantly reducing caspase activation in human endometrial stromal cells (hESCs) *in vitro* (Marshall *et al.*, 2011). DHT signalling via ARs also has a role in gland development, stromal–epithelial interactions, cell cycle and angiogenesis-related pathways in human (Marshall *et al.*, 2011) and rodent (Nantermet *et al.*, 2005; Choi *et al.*, 2015; Simitsidellis *et al.*, 2016) endometrium.

### Decidualization and endometrial receptivity

As with many of the processes in reproductive biology discussed so far, while the mid-secretory decidualization of endometrial stromal cells is primarily driven by estrogen and progesterone (Gellersen and Brosens, 2014), androgens may also play a role (Gibson *et al.*, 2016a). Indeed, plasma androgen levels, particularly androstenedione, testosterone, and DHT, are elevated in the early secretory phase (Fig. 3) (Dawood and Saxena, 1976), accompanied by elevated local testosterone, DHEA, and androstenedione production in the endometrium itself (Guerrero *et al.*, 1975). The decidualized mouse endometrium also expresses steroidogenic enzymes (Das *et al.*, 2009), facilitating elevated androgen levels, at least at the tissue level, persisting into the second half of the estrous cycle (Fig. 3).

The surface of the endometrium also undergoes adaptation in the secretory phase, becoming receptive for a brief window of time. This transformation is driven by estrogen, owing to both ovarian and local tissue androgen metabolism (Hausknecht *et al.*, 1982; Ma *et al.*, 2003), and progesterone (Young, 2013). DHEA has been shown to contribute to endometrial receptivity (Qin *et al.*, 2016) and decidualization (Çelik *et al.*, 2017), even with advanced reproductive age (Gibson *et al.*, 2018b). Secretory phase androgen activity may help initiate estrogen-driven endometrial immune regulation and vascular remodelling in preparation for pregnancy (Gibson *et al.*, 2013; Simitsidellis *et al.*, 2016). Furthermore, experiments manipulating murine decidual endometrial stromal cells via DHEA treatment demonstrate these processes are AR-dependent (Qin *et al.*, 2016).

Evidence from hESCs shows that androgen treatment *in vitro* promotes cellular transformation, increasing decidualization markers, such as prolactin and Ig-binding protein 1, and regulating cell motility and cytoskeletal organization (Kajihara *et al.*, 2012; Gibson *et al.*, 2018b). Both excess androgens and inadequate androgen signalling in hESCs *in vitro* have adverse effects on cell migration, proliferation, and endometrial receptivity (Gibson *et al.*, 2016b; Rahman *et al.*, 2018). Furthermore, insufficient androgen signalling in mice resulted in delayed implantation, while androgen excess was associated with early pregnancy loss (Diao *et al.*, 2008; Sharma *et al.*, 2021; Zhang *et al.*, 2021). Decidualization and vascular development in mice could also be blocked by inhibiting aromatase (Das *et al.*, 2009), thus disrupting the androgen: estrogen balance (Gibson *et al.*, 2016b). Aberrant or insufficient androgen signalling during decidualization can result in a transcriptional profile that is ‘out of phase’ with normal blastocyst and endometrial activity, which can affect endometrial receptivity and blastocyst implantation (Gibson *et al.*, 2016a,b).

## Abnormal androgen signalling

### Polycystic ovary syndrome

Much of our understanding regarding the role of androgen signalling in the ovaries and endometrium has come from patients with PCOS, a heterogeneous and multifactorial endocrine condition that affects between 6% and 20% of women of reproductive age (Bozdag *et al.*, 2016; Witchel *et al.*, 2019). PCOS is characterized by symptoms including hyperandrogenism, menstrual irregularities, and a polycystic ovarian morphology; the current consensus for diagnosis requires at least two of these three symptoms (Rotterdam ESHRE/ASRM-Sponsored PCOS Consensus Workshop Group, 2004; Teede *et al.*, 2018). Patients with PCOS that includes hyperandrogenism, particularly elevated circulating free testosterone (Ilic *et al.*, 2016; Zhou *et al.*, 2017; Franik *et al.*, 2019; Grassi *et al.*, 2020) and androstenedione (Georgopoulos *et al.*, 2014), may experience acne, ovarian dysfunction. infertility, and hirsutism (Escobar-Morreale, 2018; Pfieffer, 2019). In some patients, decidualization is altered, and genes relating to mitochondrial function and progesterone signalling are differentially expressed (Khatun *et al.*, 2021).

At the neuroendocrine level, persistently elevated GnRH pulsatility and an altered LH:FSH ratio contribute to elevated androgen synthesis and consequentially impaired follicular development (McCartney and Campbell, 2020; Stener-Victorin *et al.*, 2020; McCartney *et al.*, 2022). Within the ovary, the balance between androgens, anti-Müllerian hormone (AMH), and FSH is disrupted, leading to follicular arrest (Franks *et al.*, 2008). Elevated LH promotes androgen production in theca cells, while insufficient FSH and androgen metabolism to estradiol results in failure to select a dominant follicle, causing anovulation (Franks *et al.*, 2008; Franks and Hardy, 2020). Furthermore, elevated AMH, often seen in PCOS patients, inhibits the transition of primordial to primary follicles, and the increased growth of small follicles combined with growth arrest results in a polycystic morphology (Rosenfield and Ehrmann, 2016). Elevated AMH levels in the brain further stimulate the HPG axis and promotes downstream androgen production (Silva and Giacobini, 2021). There is a perpetual state of hyperandrogenism, as elevated circulating androgens suppress steroid hormone-binding globulin (SHBG) (Rasquin Leon *et al.*, 2022), and disrupted estrogen, progesterone negative feedback loops further enable LH hypersecretion (McCartney *et al.*, 2022). Furthermore, serum SHBG levels are reduced, such that androgen levels are not only elevated, but bioavailability of androstenedione, testosterone, and DHT is also increased (Xing *et al*., 2022a).

Theca cells, responsible for ovarian androgen synthesis, have an altered phenotype in PCOS. A number of recent articles reveal associations between altered androgen steroidogenesis pathways, PCOS and hyperandrogenism, including 17α-hydroxylase/17,20-desmolase (CYP17) gene polymorphisms (Heidarzadehpilehrood *et al.*, 2022; Xing *et al.*, 2022a,b), and increases in 21-hydroxylase, 11β-hydroxylase, 17α-hydroxylase, and 3α-HSD activity (Marti *et al.*, 2017; Dhayat *et al.*, 2018). Elevated 5α-reductase expression (Wu *et al.*, 2017), specifically in the context of the backdoor pathway, with associated urinary increases in 11-hydroxy androgen metabolites (Blumenfeld *et al.*, 2016; Dhayat *et al.*, 2018), has also been identified.

A small proportion of elevated androgens in PCOS patients may result from activation of backdoor synthesis pathways, as evidenced by elevated 17-hydroxy progesterone (17-OHP) and 11-oxygenated androgens (O’Reilly *et al.*, 2017; Dhayat *et al.*, 2018). However, Saito *et al.* (2016) demonstrated that the increase is instead primarily from conventional pathways (SaIto *et al.*, 2016), with the most notable increase in patients with PCOS being DHEA (Dhayat *et al.*, 2018). Indeed, both supplemental DHEA and DHT have been used to induce PCOS-like symptoms in animal studies (Mahmoud *et al.*, 2018; Bracho *et al.*, 2020; Stener-Victorin *et al.*, 2020; Corrie *et al.*, 2021; Vojnović Milutinović *et al.*, 2021; Kamada *et al.*, 2022). Elevated prenatal testosterone contributes to a metabolic and neuroendocrine phenotype consistent with PCOS (Amalfi *et al.*, 2012; Abbott *et al.*, 2019), as observed in humans (Risal *et al.*, 2019) and demonstrated in animal models (Stener-Victorin *et al.*, 2020).

Despite androgen excess, PCOS in humans is associated with reduced ovarian AR expression (Gao *et al.*, 2020), indicative of alternative signalling through ERs, either directly or via aromatization (Aflatounian *et al.*, 2020; Walters, 2020). Interestingly, studies in mice have shown that inactivating AR signalling in ovarian theca cells only partially prevented the development of hyperandrogenism-associated ovulatory dysfunction and PCOS-like features (Caldwell *et al.*, 2017; Ma *et al.*, 2017). This indicates that ovarian AR-mediated androgen actions are important but perhaps not critical in mediating the development of PCOS traits; there may also be an extraovarian mediator (Caldwell *et al.*, 2017). Upregulated steroidogenic capacity (Magoffin, 2007) and increases in androstenedione, testosterone, and DHEA levels (Benjamin *et al.*, 2021) may be in part compensating for the reductions in ovarian AR expression in patients with PCOS (Gao *et al.*, 2020). Endometrial AR expression, on the other hand, is increased in PCOS (Apparao *et al.*, 2002), and some research shows endometrial GPER expression is also elevated (Hulchiy *et al.*, 2016).

### Trans men

In addition to PCOS, the effects of elevated androgens in the ovaries and endometrium are seen in trans men receiving gender-affirming hormonal therapy. Sustained elevated testosterone levels in trans men is frequently associated with multi-follicular ovaries, elevated endometrial cell proliferation, and myometrial hypertrophy, with elevated myometrial AR expression (Loverro *et al.*, 2016), as well as either a proliferative or atrophic endometrium, depending on whether ovaries (and therefore estrogen production) are also retained (Hawkins *et al.*, 2021). While studies on PCOS and trans male patients receiving testosterone therapy demonstrate the effects of androgen excess, or levels higher than the female biological ‘norm’, the roles and regulations of androgens in the normal female reproductive tract are still not well-known.

### Cancer

Information from ovarian cancer has also increased our knowledge on steroid hormone signalling in ovarian biology. AR overexpression in ovarian cancer cells *in vitro* resulted in the downregulation of tumour suppressor genes and increased cell growth, migration, invasion, and renewal capacity (Zhu *et al.*, 2017; Mizushima and Miyamoto, 2019; Chung *et al.*, 2021). Similarly, metastatic endometrial cancer lesions express elevated AR (Tangen *et al.*, 2016; Abu Shahin *et al.*, 2021). When it comes to steroidogenesis in female reproductive cancers, 3β-HSD activity has been identified in both ovarian tumours and malignant endometrial cells (Papacleovoulou *et al.*, 2009), and increased expression of aromatase, 3β-HSD, and 17β-HSD enzymes is known to help create a pro-estrogenic environment in endometrial cancer (Segawa *et al.*, 2005; Papacleovoulou *et al.*, 2009; Cornel *et al.*, 2012; Ito *et al.*, 2016). Furthermore, 5α-reductase expression in endometrial tumours may promote local DHT synthesis (Tanaka *et al.*, 2015).

### Endometriosis

Beyond cancers, androgens and altered AR expression have also been implicated in endometriosis (Carneiro *et al.*, 2008; Park *et al.*, 2014; Babayev *et al.*, 2017; Lv *et al.*, 2022). It has been suggested that endometriosis is associated with low prenatal testosterone exposure and low postnatal testosterone levels, as well as low LH relative to FSH, and increased levels of SHBG and aromatase (Bulun *et al.*, 2004; Maia *et al.*, 2012, 2009; Ono *et al.*, 2014; Dinsdale *et al.*, 2021; Dinsdale and Crespi, 2021). Through the complex network of steroid hormone receptor regulation (Fig. 2), androgens may be responsible for mediating altered estrogen signalling and PR functionality in this estrogen-dominant, progesterone-resistant condition (Babayev *et al.*, 2017), and have also been linked to endometriosis-associated chronic pain (Evans *et al.*, 2021). Further detail on these pathologies and the role of androgens is described elsewhere (Gibson *et al.*, 2018a; Lv *et al.*, 2022).

### Menopause

Menopause is initially associated with a relative increase in the levels of circulating androgens (a result of decreased estrogen and SHBG), but is coupled with ovarian and adrenal ageing, and a decline in steroidogenesis (Brzozowska and Lewiński, 2020). Reduced sexual desire in post-menopausal women is well documented, and may be linked to androgen levels (Sarrel, 1999; Nazarpour *et al.*, 2019). In addition to its effects in endometrial and ovarian physiology discussed in this review, the potential for testosterone supplementation in post-menopausal women to improve symptoms of female sexual dysfunction has recently been discussed (Jayasena *et al.*, 2019; Nappi, 2019; Ingram *et al.*, 2020), highlighting a potential new focus on the role of androgens in female sexual health.

### Monogenic disorders of androgen synthesis

Monogenic disorders of androgen synthesis also demonstrate the effects of androgen insufficiency, such as androgen insensitivity syndrome (AIS), caused by AR dysfunction. AIS is a difference in sexual development, typically diagnosed in individuals with an XY karyotype consistent with male, with underdeveloped testes and under-masculinized or even female-appearing external genitalia (Mongan *et al.*, 2015; Baranowski *et al.*, 2018). In XX individuals, monogenic disorders like AIS involve disruptions to many elements of the androgen synthesis pathway, including 21-hydroxylase and 11β-hydroxylase deficiencies, impaired activity of 3β-HSD2, and poor ovarian response (Baranowski *et al.*, 2018; Finkielstain *et al.*, 2021). While AIS primarily affects adrenal steroidogenesis, mutations in aromatase, as well as cholesterol cleavage enzymes StAR and *CYP11A1*, can result in primary ovarian insufficiency (Naamneh Elzenaty *et al.*, 2022). Alterations in other steroidogenic enzymes have been identified in XY individuals (Baranowski *et al.*, 2018; Finkielstain *et al.*, 2021); however, much of this pathway in XX females is still unexplored, even in the normal state.

## Limitations and considerations for androgen research

It is clear that androgen signalling is incredibly complex. Androgens are synthesized from several different pathways, can be converted to more potent forms as well as to estrogens, and interact with a range of receptors (Fig. 2). Steroid hormone receptor expression patterns across the menstrual cycle are well documented. However, the effects of DHEA and androstenedione may be both direct and indirect via conversion to other androgens and more active metabolites with higher binding affinities (Prough *et al.*, 2016). Thus, there is a gap in the literature regarding specific receptor interactions that are responsible for androgen effects universally, but particularly in females.

Much of our understanding of androgens in the female reproductive system comes from PCOS, studies using rodent models, and the effects of hormonal replacement therapy for either transgender men (Miller *et al.*, 1986; Loverro *et al.*, 2016; Hawkins *et al.*, 2021) or patients with poor ovarian response (Balasch *et al.*, 2006; Li *et al.*, 2015; Chern *et al.*, 2018). These studies used exogenous androgens above normal physiological levels; thus, there remain significant gaps in our understanding regarding the role of androgens, particularly recently identified androgen subtypes (11KA4, 11KT, and 11KDHT), in the normal state.

ARKO animal models are used extensively to demonstrate abnormal ovarian function and reduced fertility (Hu *et al.*, 2004; Shiina *et al.*, 2006; Sen and Hammes, 2010; Ma *et al.*, 2017), and abnormal uterine growth (Walters *et al.*, 2009; Zhou, 2010). However, our understanding of AR functions from these models may depend on whether a global AR knockout (Hu *et al.*, 2004; Shiina *et al.*, 2006; Walters *et al.*, 2017) or tissue-specific knockout model (Sen and Hammes, 2010; Chang *et al.*, 2013; Ma *et al.*, 2017) is used (Zhou, 2010), as well as the suppression of normal hormones (Zeleznik *et al.*, 2004) or ovariectomy (Simitsidellis *et al.*, 2016) prior to exogenous hormone treatment. These elements and their effect on existing androgen levels, and subsequently interactions with other hormones, may be important to consider given the complex signalling networks discussed in this review.

The effects of androgen treatment, such as hormone replacement therapy in humans, will vary significantly based on the type, quantity, method, and duration of androgens administered. Individual sensitivity to the hormones will also need to be accounted for (Lu, 1998). For example, a study that investigated androgen-based hormone replacement therapy, in which serum testosterone levels were monitored and maintained at a consistent level, reported no endometrial atrophy (Loverro *et al.*, 2016). In contrast, others did not account for individual differences in testosterone uptake and metabolism, and reported endometrial atrophy and reduced cell proliferation, with lower levels of hormones administered, over a similar time period (Miller *et al.*, 1986; Perrone *et al.*, 2009).

Additionally, observed androgen levels will vary greatly depending on the choice of sample collected (blood, saliva, or tissue), the choice of assays for analysis, and timing of sample collection (both time of day and stage of the menstrual cycle). Perhaps the most important consideration is the bioavailability of testosterone, which may or may not be accounted for, depending on whether free testosterone (Ostrowska *et al.*, 1998; Rothman *et al.*, 2011), SHBG-bound testosterone, or total testosterone levels (Vermeulen and Verdonck, 1976) are investigated (Simons *et al.*, 2021). Some evidence suggests a peak in free testosterone in the proliferative phase, and a decrease in total testosterone levels in the secretory phase (Bloch *et al.*, 1998). With the effect of SHBG acknowledged, it appears that DHT does not fluctuate across the menstrual cycle (Rothman *et al.*, 2011), while others measuring total plasma levels have instead reported significant cyclic variations in androstenedione, testosterone, and DHT (Vermeulen and Verdonck, 1976). Given that binding proteins reduce the bioavailability of androgens, a ratio of free to bound androgen may be more accurate (Laurent *et al.*, 2016), though not currently used extensively in the literature.

Additionally, detection methods that are commonly used may not accurately represent the entire functional pool of androgens. It is well reported that traditional detection methods (i.e. immunoassays) lack the sensitivity required for all relevant androgen subtypes in comparison to liquid chromatography-tandem mass spectrometry, particularly for c11-oxy adrenal androgens (Demers, 2010; Tosi *et al.*, 2016; Grassi *et al.*, 2020; Caron *et al.*, 2021). Highly specific methods are now being used more often, but much of our understanding of androgens in females to date relies on studies that have utilized traditional detection methods, which has significant limitations when it comes to investigating androgen levels, functionality, and regulation.

It is also important to consider local production and metabolism of androgens in tissues such as the endometrium. Androgen levels in peripheral blood will not entirely reflect the total presence, activity, conversion, or cyclic differences in androgens in a way that is biologically relevant (Schiffer *et al.*, 2018). For example, in the secretory phase, progesterone stimulates 17β-HSD activity, increasing the conversion of androstenedione to testosterone in endometrial tissue (Hausknecht *et al.*, 1982), while serum testosterone levels tend to be relatively low and stable in the secretory phase, following a peak around the time of ovulation (Atukorala *et al.*, 2022; Krüger *et al.*, 2023). Many publications do not acknowledge the potential effects of local androgen synthesis, nor the contributions of other ‘non-classical’ androgen subtypes.

## Conclusion

Overall, the evidence to date supports that androgens and their receptors have a significant role to play in female reproductive and sexual health. As we have discussed in this review, there are a number of necessary regulatory mechanisms in place in the female reproductive tract that would allow for androgens to be produced locally, such as the expression of steroidogenic enzymes. There is also evidence for the role of androgens in follicular development, decidualization, and implantation. It is therefore important to design studies specifically investigating these roles to get a complete picture of androgen activity in normal physiology (Table 3).

**Table 3.** Future research questions.

Future research questions: What are the specific effects and regulation of androgens throughout follicular development and the ovarian cycle? How do androgens impact follicular estrogen production? How is the influence of testosterone replaced by estrogen as the dominant follicle develops? What roles do androgens play in decidualization and endometrial cell differentiation? What roles do 11-oxy androgens produced in alternative synthesis pathways have in the normal endometrium and ovaries? How does our knowledge of the role of androgens in ovarian and endometrial biology change when we consider the contributions of numerous androgen subtypes, synthesis pathways, and complex receptor interactions? How does this impact our knowledge and use of androgens clinically?

In female reproductive health and research, often androgens are not taken into account, or are only properly considered when it comes to their abnormal levels or regulation in disorders. Our understanding of the complex array of androgens in females has also been limited by our ability to measure and quantify the subtypes, and only recently have more androgens been identified, likely with important physiological roles. Thus, to conclude this review, we re-emphasize the need for more comprehensive studies on androgens in female physiology (Table 3), taking into account all variables that affect androgen levels and activity, such as the source and timing of samples, presence of SHBG, and receptor interactions. Measuring one subtype alone and extrapolating for androgens as a whole is highly likely to mislead us.

## Data Availability

No new data were generated or analysed in support of this article.
